# Supplementary material for: PEGylation Prolongs the Half-Life of Equine Anti-SARS-CoV-2 Specific F(ab’)2
Source: Int J Mol Sci. 2023 Feb 8;24(4):3387. doi: 10.3390/ijms24043387 (PMC9963672; doi:10.3390/ijms24043387)
Supplement: Supplementary file 1 [file ijms-24-03387-s001.zip › ijms-2119420-supplementary.pdf]

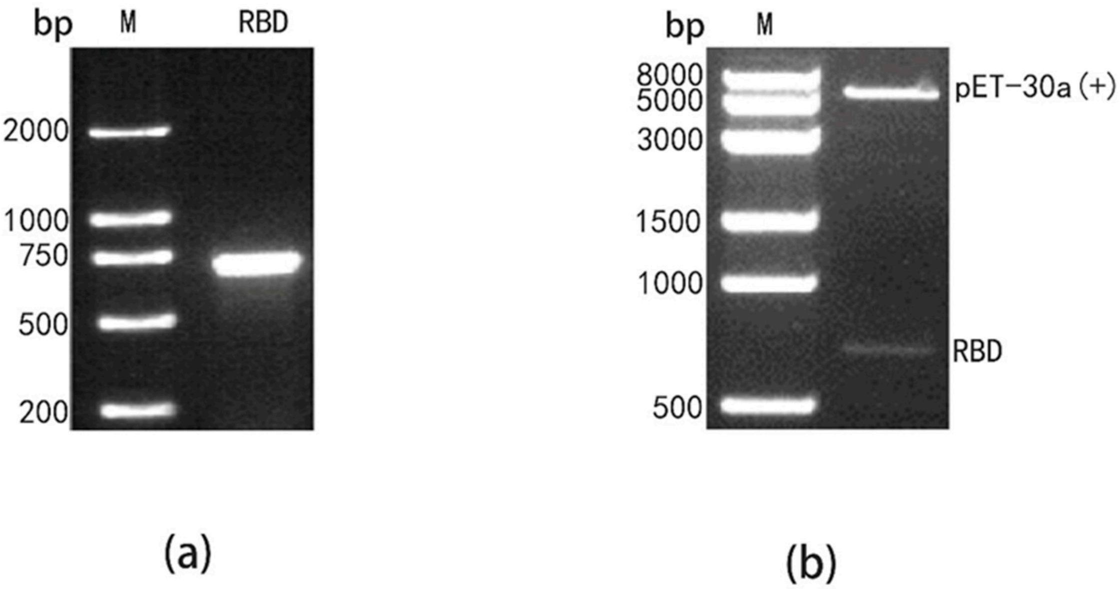


**Figure S1.** (**a**) PCR identification results of RBD gene. (**b**) PCR identification of recombinant expression plasmid PET-30a(+)-RBD.
